# The COP1 E3-ligase interacts with FIP200, a key regulator of mammalian autophagy

**DOI:** 10.1186/1471-2091-14-1

**Published:** 2013-01-06

**Authors:** Saori Kobayashi, Noriko Yoneda-Kato, Nagisa Itahara, Akihiro Yoshida, Jun-ya Kato

**Affiliations:** 1 Graduate School of Biological Sciences, Nara Institute of Science and Technology, Nara, Japan

**Keywords:** COP1, FIP200(RB1CC1), UV, Autophagy

## Abstract

**Background:**

The ubiquitin ligase COP1, COnstitutively Photomorphogenic 1, functions in many biological responses in mammalian cells, but its downstream pathway remains unclear.

**Results:**

Here, we identified FIP200, a key regulator of mammalian autophagy, as a novel COP1-interacting protein by yeast two-hybrid screening. The interaction was confirmed by a GST-pulldown assay. Split-GFP analysis revealed that interaction between COP1 and FIP200 predominantly occurred in the cytoplasm and was enhanced in cells treated with UV irradiation. Different forms of FIP200 protein were expressed in cultured mammalian cells, and ectopic expression of COP1 reduced one of such forms.

**Conclusions:**

These data suggest that COP1 modulates FIP200-associated activities, which may contribute to a variety of cellular functions that COP1 is involved in.

## Background

COP1, COnstitutively Photomorphogenic 1, is the ubiquitin ligase containing RING-finger, Coiled-coil and WD40 domains [1,2], and well conserved from plants to animals [2]. In plants, COP1 was identified as one of the COP proteins that act as a repressor of photomorphogenesis [1], and functions downstream of the COP9 signalosome complex [1-3] as a component of a multimeric E3 ubiquitin ligase complex that includes Cullin 4 (CUL4), Damaged DNA-Binding Protein 1 (DDB1), RING-Box 1 (RBX1), and Suppressor of Phya (SPA) proteins [4]. In response to multiple plant photoreceptors, the COP1-CUL4-DDB1-RBX1-SPA complex controls many light-regulated transcription factors [2,5].

In contrast to its specific role in plants, mammalian COP1 is involved in many biological responses such as tumorigenesis [6-9], DNA damage response [10,11], lipid metabolism [12], and gluconeogenesis [13] by targeting different substrates for degradation, which include p53 [6], c-Jun [8,14], Ets1/2 [9], TRB3 [12], and TORC2 [13]. Particularly, in a DNA-damage responsive pathway, COP1 functions downstream of ATM/ATR kinases by direct phosphorylation [10,11], but the precise mechanism remains to be determined. Considering a wide range of COP1 action in various biological responses, components and pathways downstream of COP1 are not fully understood yet.

To better understand the COP1-signaling pathway, we searched for novel COP1-interacting proteins by yeast two-hybrid screening and identified FIP200 as one such candidate. FIP200 (also known as RB1-inducible Coiled-Coil 1, RB1CC1) was first reported as a regulator of the retinoblastoma (RB) protein [15], identified as a tumor suppressor in human breast cancer [16,17], and recently rediscovered as a mammalian counterpart of Atg17 in the yeast Atg1-Atg13-Atg17 complex [18]. The mammalian ULK1(Atg1)-Atg13-FIP200(Atg17) complex functions downstream of mTOR, and, together with the Beclin 1-Vps34 kinase pathway and the Atg5-Atg12 and LC3 conjugation systems, plays a key role in the induction of autophagy, an intracellular lysosomal degradation system for cytoplasmic proteins and organelles [19-23].

In this study, we investigated the interaction between COP1 and FIP200 by the yeast two-hybrid assay, the GST-pulldown assay, and the Split-GFP assay. Proliferating mammalian cells expressed several different forms of FIP200 protein, and one of them was downregulated by the ectopic overexpression of COP1 protein, suggesting that COP1 modulates FIP200-associated biological activities in a certain occasion, which may contribute to the complexity of the COP1-associated function.

## Results

### Identification of FIP200 as an interactor with COP1

To explore the novel signaling pathway mediated by COP1, we sought a candidate for interactors with COP1 by yeast two-hybrid screening of the human K562 erythroleukemia cDNA library. Out of 1.6 × 10^6^ transformants, we chose 13 potential clones that repeatedly exhibited positive signals. These clones contained part of two independent cDNAs, one for Jun D and one for FIP200 [24]/RB1-inducible Coiled-Coil 1 [15] (RB1CC1). The presence of the former cDNA was anticipated given that c-Jun is a substrate of COP1 [14,25] and that JunD is highly homologous to c-Jun, both of which belong to the same family of AP1 transcription factors. The latter component, FIP200, also termed RB1CC1, was originally shown to control retinoblastoma protein [15] and functions as a tumor suppressor in human breast cancer [16]. FIP200 was recently rediscovered as a component of the mammalian ULK1 (Atg1)-Atg13-FIP200 (Atg17) complex and plays an important role in the induction of autophagy [18]. Therefore, we decided to investigate the COP1-FIP200 interaction and the role of COP1 in terms of UV response and induction of autophagy.

**Figure 1:**
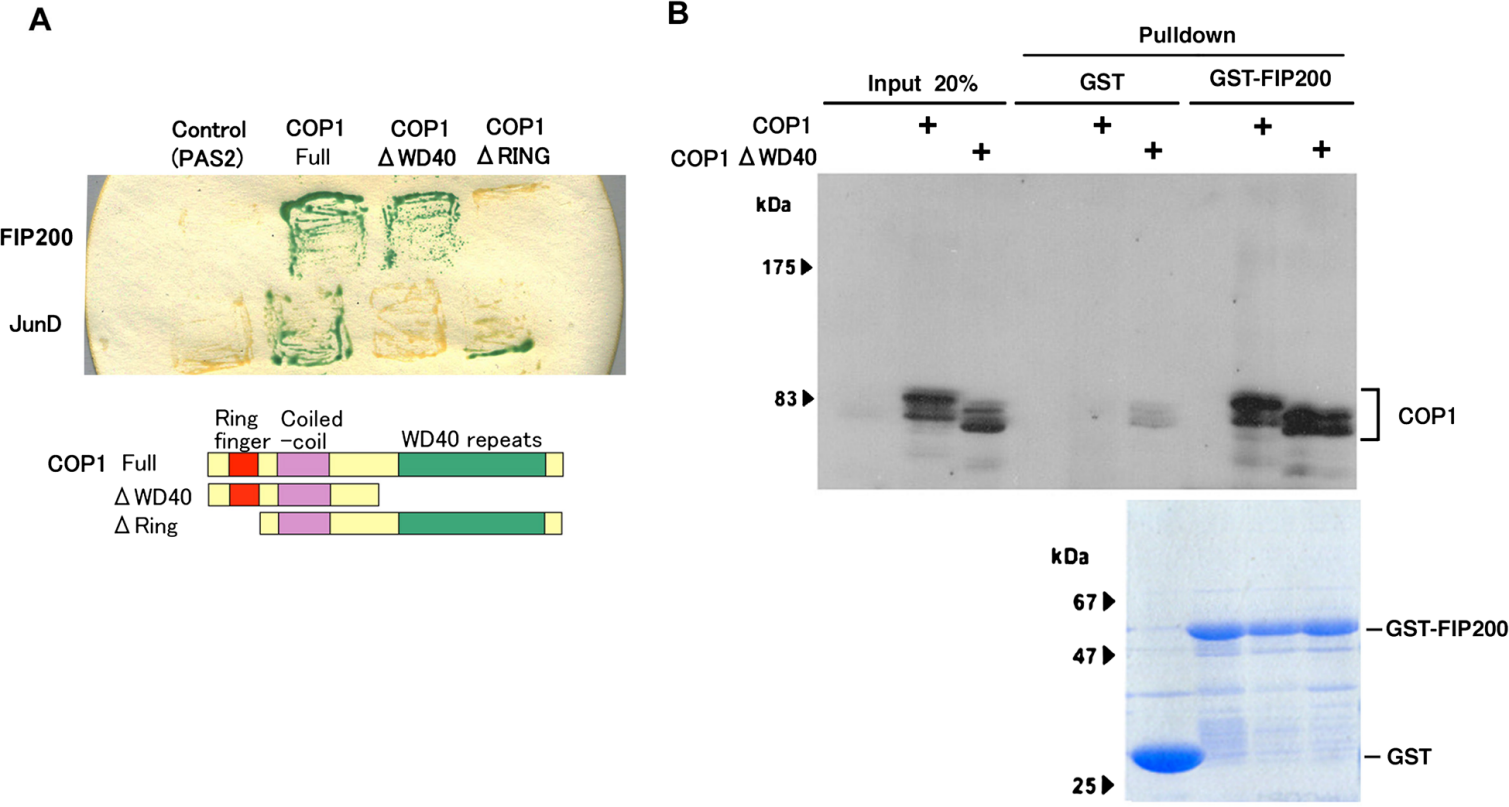
**FIP200 interacts with COP1. (A)** Y190 yeast cells were transfected with pAS2 vectors containing full-length and deletion mutants (ΔWD40 and ΔRING) of COP1 together with pACT vectors containing FIP200 and JunD. Transfectants were grown on a Trp^-^ Leu^-^ His^-^ selection medium and tested for β-galactosidase activity (top). Schematic structure of COP1 mutants (bottom). **(B)** GST and GST-FIP200 recombinant proteins were immobilized on glutathione beads (bottom panel, separated by SDS-PAGE and stained with CBB) and mixed with cell lysate containing ectopically expressed HA-tagged wild-type and ΔWD40-mutant form of COP1. Bound proteins were analyzed by Western blotting using antibody against COP1 (upper panel).

A yeast two-hybrid analysis using deletion mutants of COP1 (Figure 1A) indicated that the RING domain at the N-terminus of COP1 [6], but not the WD40 domain, is required for interaction with FIP200, showing a clear difference from JunD, which interacted with the WD40 domain as is the case with most substrates for ubiquitin ligases containing the WD40 motif [2]. In vitro binding assays using GST-fused FIP200 protein (Figure 1B) and cell lysate containing the ectopically expressed HA-tagged COP1 (wild type and a mutant lacking the WD40 domain) showed that COP1 and FIP200 interacted in vitro (Figure 1B).

### Different forms of FIP200 protein were expressed in cultured mammalian cells

**Figure 2:**
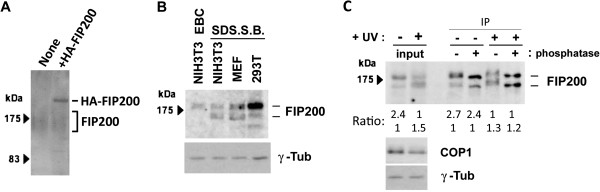
**Different forms of FIP200 protein were expressed in cultured mammalian cells. (A)** HEK293T cells were mock-transfected (None) and transfected with an expression vector containing HA-tagged FIP200. Cell lysates were analyzed by Western blotting using antibody against FIP200. **(B)** NIH3T3, MEF, and 293T cells were lyzed in EBC and SDS sample buffers and analyzed by Western blotting using antibodies against FIP200 and γ-tubulin. **(C)** NIH3T3 cells were treated with UV then incubated in complete medium, and lyzed in SDS sample buffer (input). Cell lysates in SDS sample buffer were diluted with EBC buffer, and FIP200 protein was immunoprecipitated with antibody against FIP200, treated with phosphatase, and visualized by Western blotting using antibody against FIP200. Cell lysates were also analyzed by Western blotting using antibodies against COP1 and γ-tubulin. The ratio between upper and lower bands of FIP200 is also shown at the bottom of the FIP200 blot.

To analyze the function of FIP200 in mammalian cells, we raised a rabbit polyclonal antibody to FIP200 using a polypeptide corresponding to the region isolated by the yeast two-hybrid screening, which specifically reacted with endogenous FIP200 as well as ectopically-expressed FIP200 protein by Wester blotting (Figure 2A). In the lysate isolated from proliferating mammalian cells, our antibody recognized two forms of FIP200 (Figure 2B), the slower migrating form being more readily extracted from the cells (EBC buffer containing 0.5% NP40 versus SDS sample buffer containing 1% SDS). Because we have previously showed that COP1 is involved in cellular response mediated by UV stimulation [11], we examined whether UV might affect FIP200. Interestingly, UV stimulation altered the ratio between these two forms (Figure 2C); proliferating cells contained the slower migrating form more, while UV treatment decreased the expression of the slower migrating form and, instead, increased that of the faster migrating form (the level of COP1 declined after UV stimulation as previously reported [11]).

FIP200 is known to be modified by phosphorylation [26], which often affects mobility in SDS-PAGE. To test this possibility, we extracted the protein from cells treated with UV and un-treated cells in an SDS sample buffer, isolated FIP200 by immunoprecipitation, and treated it with phosphatase in vitro. The result (Figure 2C) showed that the difference in mobility was not due to the level of phosphorylation although both forms were phosphorylated. Currently, we do not know the exact molecular identity of these two variants, which might be generated by alternative splicing or other post-translational modifications.

### FIP200 interacts with COP1 in the cytoplasm of proliferating cells in response to UV stimulation

We have so far not been successful in detecting the COP1-FIP200 complex in cell lysate by immunoprecipitation/immunoblotting. One possible explanation for this is that our antibody does not recognize the complex. Another possibility is that the COP1-FIP200 complex may not be efficiently eluted from the cells in a buffer suitable for immunoprecipitation. In fact, we identified different forms of FIP200 by Western blotting possibly due to alternative splicing [16] and one of them was not efficiently extracted in a buffer for immunoprecipitation (Figure 2B).

**Figure 3:**
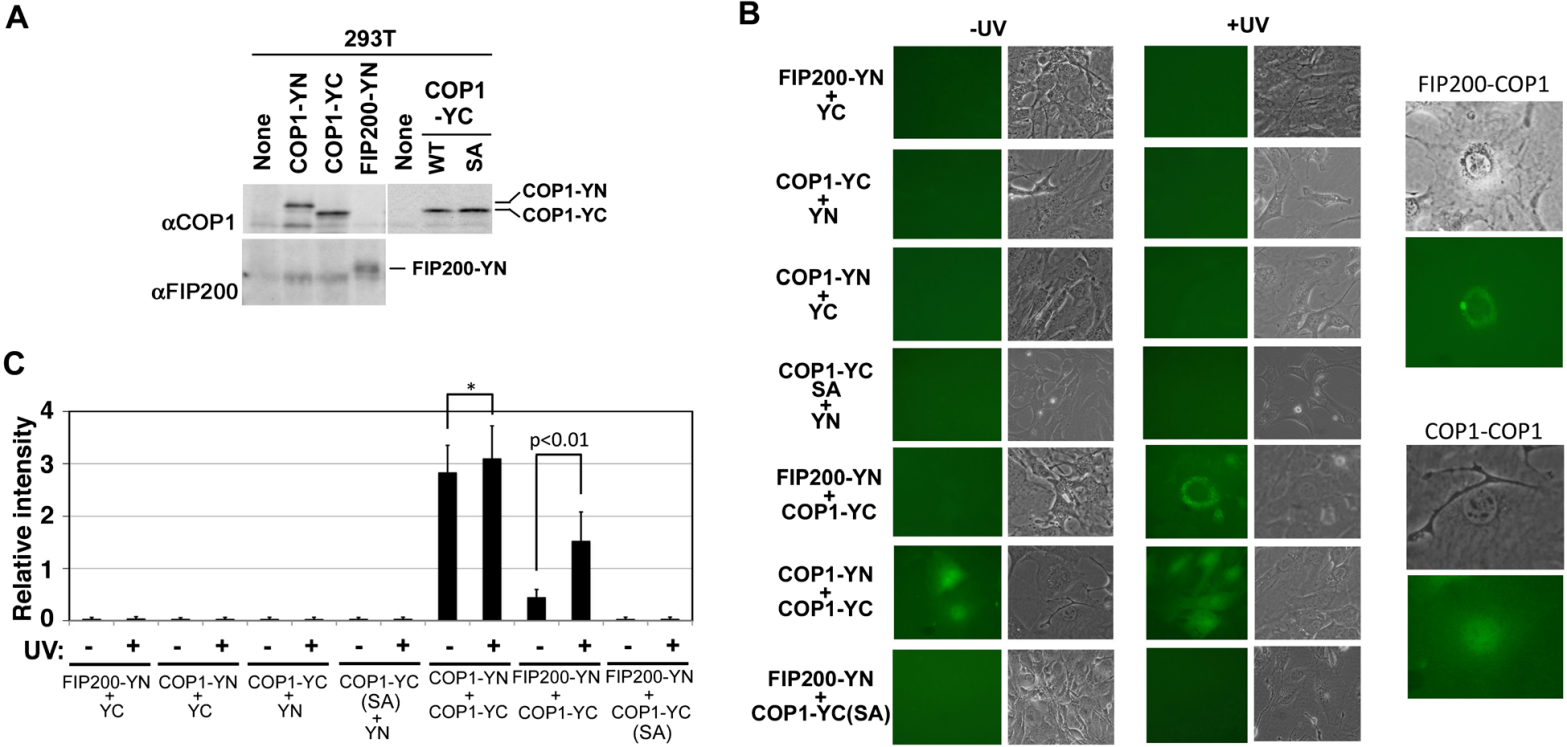
**Analysis of FIP200-COP1 interaction by Split GFP assay. (A)** 293T cells were transfected with expression vectors encoding COP1 (wild type (WT) and phosphorylation-defect mutant (SA)) and FIP200 fused with N-terminal (YN) and C-terminal (YC) halves of YFP. Cell lysates were analyzed by Western blotting using antibody against FIP200. **(B, C)** NIH3T3 cells were transfected with expression vectors encoding N-terminal (YN) and C-terminal (YC) halves of YFP alone and fused with COP1 (wild type and SA mutant) and FIP200. Before and after treatment with UV, GFP signals were observed using phase-contrast and fluorescence microscopy **(B)** and measured with a flow cytometer **(C)**. Higher magnification of photos of FIP200-COP1 and COP1-COP1 interactions are also shown (B, right panels). *; not significantly different (C).

To overcome these problems and to further investigate the interaction between COP1 and FIP200 in vivo, we performed a Split-GFP analysis [27], in which GFP (YFP) was split into two domains, N-terminal (YN) and C-terminal (YC), and fused to two molecules (COP1 and FIP200 in this case), respectively (Figure 3A). If these two molecules interact with each other in the cell, the GFP (YFP) signal will be restored. In transfected cells, COP1-YN, COP1-YC (both wild-type and mutant forms) and FIP200-YN together with YC and YN did not generate any significant signals above the background level, but we detected the GFP signal in the cells co-transfected with both wild-type COP1-YC and FIP200-YN after UV exposure (Figure 3B, C). The restored signal was predominantly in the cytoplasm (mostly perinuclear) with some in the nucleus too. We also detected interaction between COP1-YN and COP1-YC as a control. In this case, the signal was both in the nucleus and in the cytoplasm (Figure 3B). Quantification by flow cytometric analysis showed that the COP1-COP1 interaction was constitutive, whereas the COP1-FIP200 interaction was inducible in response to UV treatment (Figure 3C). Importantly, interaction was diminished when we used a COP1 mutant (SA), which contains a serine to alanine substitution at the conserved ATM/ATR phosphorylation site at the 389th codon [10] (Figure 3B, C), suggesting that UV-mediated phosphorylation of COP1 is required for the efficient formation of a complex between COP1 and FIP200. Taken together, while COP1 stably forms a multimeric complex in the cell, its binding to FIP200 in the cytoplasm is enhanced by UV stimulation.

### Ectopic expression of COP1 reduced the expression of a certain form of FIP200 protein and exhibited tumorigenisity in response to UV

**Figure 4:**
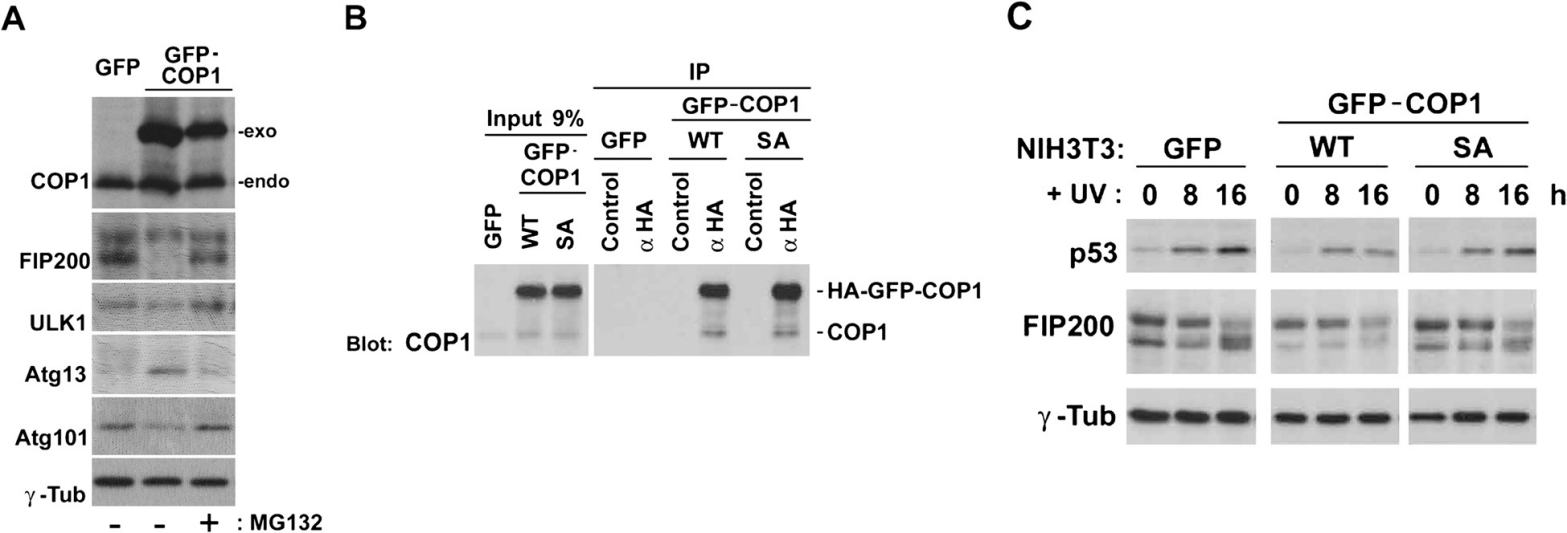
**Ectopic expression of COP1 reduced a form of FIP200 protein. (A)** NIH3T3 cells were transfected with expression vectors encoding HA-GFP and HA-GFP-fused COP1. After selection, cells were treated or non-treated with MG132, harvested, and analyzed by Western blotting using antibodies against COP1, FIP200, ULK1, Atg13, Atg101, and γ-tubulin. **(B)** NIH3T3 cells were transfected with expression vectors encoding HA-GFP and HA-GFP-fused COP1 (wild type and SA mutant). Cell lysates isolated from transfectants after selection were immunoprecipitated with normal rabbit serum (Control) and rabbit antibody to an HA-epitope (α-HA). Immune complex was analyzed by Western blotting using antibody against COP1. **(C)** Cells in panel B were treated with UV then incubated in complete medium for 0, 8, and 16 hours. Cells were harvested, and analyzed by Western blotting using antibodies against p53, FIP200, and γ-tubulin.

To examine the effect of COP1 on FIP200, we ectopically expressed GFP-tagged COP1 in NIH3T3 (NIH/GFP-COP1) cells. Figure 4A shows that the level of ectopic expression was approximately the same as that of the endogenous protein. Interestingly, the faster-migrating form of FIP200 was downregulated in NIH/GFP-COP1 cells compared to that of the control (NIH/GFP) cells transfected with the control GFP-vector, whereas the slower-migrating form remained the same. Because it is known that FIP200 forms a complex with ULK1, Atg13, and Atg101 to function downstream of mTOR to induce autophagy [18,28], we investigated their expression. Figure 4A shows that ectopic expression of COP1 affected differently; ULK1 was almost unaffected, Atg13 was upregulated and Atg101 was slightly downregulated. We did not detect any direct binding of COP1 with ULK1, Atg13, and Atg101 (negative data not shown), suggesting that COP1 affects these components through interaction with FIP200. Interestingly, treatment of cells with an inhibitor to proteasome, MG132, reversed the effect of COP1 overexpression. When we investigated autophagy in these cells, however, autophagy was fully induced in response to amino acid starvation (negative data not shown). COP1 may affect other activities associated with FIP200.

Because interaction between COP1 and FIP200 was enhanced by UV stimulation and SA mutation diminished the interaction, we ectopically expressed wild type and SA mutant form of COP1 in NIH3T3 cells, and examined the effect of UV on FIP200. Figure 4B, left panel shows that both wild type and SA mutant COP1 were successfully overexpressed. Immunoprecipitation of the HA-tagged exogenous COP1 protein with the antibody to the HA epitope brought down the endogenous COP1 protein (Figure 4B, right panel), indicating that COP1 formed a dimer or a larger multimeric complex, which was expected from the split-GFP analysis (see above, Figure 3). The SA mutant retained the ability to interact with the endogenous COP1. Upon stimulation with UV (Figure 4C), the slower migrating form of FIP200 decreased and the faster migrating form increased in control cells. Overexpression of wild type COP1 reduced the level of faster migrating form at time 0 and blocked its induction by UV stimulation, whereas overexpression of SA mutant did not affect the band shift of FIP200 upon UV stimulation.

**Figure 5:**
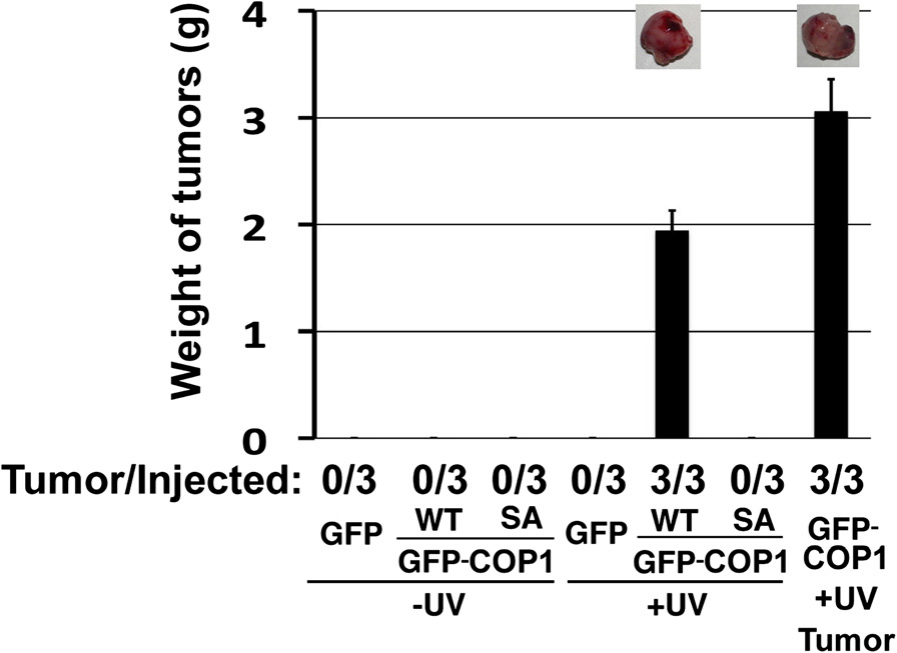
**Effect of COP1 on UV-mediated tumorigenesis.** NIH3T3 cells were transfected with expression vectors encoding HA-GFP and HA-GFP-fused COP1 (wild type and SA mutant) and selected. After exposure to UV (50 J/m^2^) and recovery culture, the cells (ca 10^6^) were subcutaneously injected into NOD-SCID mice. At 2.5 months post-injection, mice were sacrificed and the weights of the tumors were measured. Note that, except for the cells expressing the ectopic wild-type COP1 with UV treatment, no trace of the cells injected was detected. Therefore, the sizes and weights of the tumors in those cases are actually 0. Cells (ca 10^6^) recovered from the tumors were injected into NOD-SCID mice, and the size of the tumor was measured after 3 weeks (right column). The data are averages for three independent experiments. Photos of the tumors are also shown (upper).

FIP200 was identified as a tumor suppressor [16,17]. If COP1 negatively regulates FIP200, one might expect that COP1 act as an oncogene. In addition, COP1 responds to UV stimulation and becomes a substrate of ATM/ATR kinases [10,11]. We, therefore, tested whether the overexpression of COP1 facilitates cellular transformation in response to UV irradiation. We treated NIH3T3 mouse fibroblasts expressing COP1 with UV, let them recover for passaging, and subcutaneously injected them into NOD-SCID mice (Figure 5). Ectopic expression of the COP1 protein itself was not tumorigenic because no trace of cells was detected 2 months after injection. However, after treatment with UV and successive passages in a recovery culture, cells ectopically expressing COP1 formed a tumor of significant size in mice. Importantly, cells transfected with SA mutant of COP1, which did not interact with FIP200 (Figure 3B, C), failed to form tumors even after UV stimulation, suggesting that COP1 requires interaction with FIP200 to exhibit its oncogenic properties. We currently do not know the physiological significance of the impact of COP1 overexpression on autophagy. However, considering the differential effect on the expression of the components of the FIP200 complex and the FIP200 subtypes and that tumorigenic function of COP1 requires interaction with FIP200, it is feasible to say that COP1 may regulate biological activities associated with FIP200 in a certain occasion.

## Discussion

As distinct from its plant counterpart [1], mammalian COP1 is involved in many biological occasions [2]. Multi-functionality of COP1 partly stems from its variety of substrates and various adaptor or accessory proteins to interact with. Although several proteins have been identified as the target of COP1 [6,8,9,12-14], it is reasonable to speculate that more substrates and downstream pathways are yet to be found. Our findings imply that autophagy may situate downstream of the signaling pathway mediated by COP1, which may partly explain the multifunction of COP1 because autophagy is reported to be involved in many biological occasions [20,21].

By yeast-two hybrid screening, we identified C-terminal polypeptide of FIP200 as the interactor of COP1, and raised antibody against this portion of the protein. Using this antibody, we detected at least two different forms of FIP200 in proliferating mammalian cells, both of which should, therefore, share the epitope in the C-terminus of FIP200. Currently, we do not know the identity of these two forms of FIP200 recognized by our antibody because phosphorylation (Figure 2) and ubiquitination (anti-ubiquitin antibody failed to recognize either form. negative data not shown) do not seem to account for the mobility difference of these two forms. It is feasible to suspect that alternative splicing generated these two forms, but the extensive RT-PCR analysis using a series of primers designed within different exons failed to identify the alternative transcripts (negative data not shown). Therefore we do not exclude the possibility that the faster-migrating form is the product of other post-translational modifications. Whatever the mechanism, it is important to emphasize that these two forms were extracted in different conditions and that only the faster-migrating form was downregulated by the ectopic expression of COP1, suggesting that they locate in different compartments within the cell and that one of the two is the possible target of COP1.

We do not yet know whether FIP200 is a substrate for the COP1 ligase. FIP200 bound to the RING domain, but not the WD40 domain, of COP1, which makes a clear difference from other substrates such as JunD. We, so far, did not see the ubiquitinated FIP200 protein in COP1-overexpressing cells. However, we did observe the downregulation of faster-migrating form of FIP200 in COP1-overexpressing cells in an MG132-sensitive manner, suggesting that COP1 somehow induced proteasome-mediated degradation of FIP200. At present, we do not exclude the possibility that COP1 altered the level of FIP200 expression through mechanisms other than direct ubiquitination. COP1 might affect alternative-splicing to affect the expression of faster-migrating form, which step is sensitive to the action of proteasome.

Cells with ectopic overexpression of COP1 still underwent autophagy in response to amino acid starvation even though the faster-migrating form of FIP200 was efficiently downregulated, and the expression of Atg13 and Atg101 was modulated (although slightly). It could be that the remaining components of the FIP200 complex were sufficient to the initiate autophagic program or alternative form of FIP200 may respond to different inducers of autophagy such as UV. To answer this question, molecular identification of two forms of FIP200 is the urgent matter. Knowing the difference between the two, we could compare the composition of the different complexes and examine the role of each form in response to various stimuli, and the potential functions associated with FIP200 in the cell cycle control [15], p53 regulation [17] and DNA damage repair [29] as well as autophagy [18].

We have tried to establish the in vitro ubiquitination assay for COP1 and FIP200 using recombinant proteins without success (negative data not shown). This could be due to the lack of the COP1-accessory proteins or, alternatively, COP1 may favor the FIP200-containing complex rather than a single polypeptide. In addition to the identity of FIP200 variants, an adaptor protein of COP1 specific to FIP200 will be required for establishment of the in vitro reconstitution system, which will give us many clues to biochemically understand the nature of COP1-associated activities in mammals.

## Methods

### Yeast two-hybrid screening

The entire coding sequence (full) and deletion mutants (ΔWD40 and ΔRING,) of mouse COP1 were fused in-frame to the GAL4 DNA-binding domain of the pAS2 vector [30]. The resulting ‘bait’ plasmid (pAS2-COP1) was used to screen pACT- human K562 erythroleukemia libraries (library size: 3 × 10^6^, Clontech) by the yeast two-hybrid method in Y190 yeast cells [11,30,31].

### In vitro binding assay

A cDNA fragment containing the C-terminal domain of FIP200 (amino acid residues 1357–1594) was inserted into the pGEX vector (Pharmacia) in-frame with Glutathione S-transferase (GST). Expression and purification of GST-fused proteins and the binding conditions were as described [11,32].

### Cell culture, transfection, retroviral infection, and treatment with UV

NIH3T3 (Arf-null, p53-wild-type) mouse fibroblasts (provided by Drs C. J. Sherr and M. F. Roussel), mouse embryonic fibroblasts (MEFs), and 293T human embryonic kidney (HEK) cells were cultured, transfected via the calcium phosphate-DNA precipitation method [33], and infected with retroviral vectors as described [11,32,34]. For treatment with UV, cells were washed with PBS twice, exposed to UV light in a UV Crosslinker (UVP, Upland, CA) (25 or 50 J/m^2^), and incubated in a serum-containing complete medium. In some cases, cells were treated with 5 μM MG132 (Biomol, Plymouth Meeting, PA) before harvest.

### Plasmid construction

The GFP-fused protein expression vector (pMSCV-puro-GFP), into which COP1 cDNAs were subcloned, was described previously [11]. COP1 mutants (ΔWD40, ΔRING and SA) [6,10,14,35] were generated by PCR.

### Protein analyses

Cell lysis, immunoprecipitation, sodium dodecyl sulfate-polyacrylamide gel electrophoresis (SDS-PAGE), and immunoblotting were performed as described [11,34]. Two types of lysis buffer used in this study were EBC buffer (50 mM Tris–HCl pH8.0, 120 mM NaCl, 1 mM EDTA, and 0.5% NP40) containing 2000 KIU/ml of aprotinin, 1mM PMSF, 0.1 mM NaF, 0.1 mM Na3VO4, and 10 mM β-glycerophosphate, and SDS-sample buffer (40 mM Tris–HCl, pH 6.8, 0.1 M DTT, 1% SDS, 10% glycerol, and 0.05% Bromophenol Blue). In some cases, immunoprecipitated proteins were treated with phosphatase before immunoblotting [36]. A rabbit polyclonal antibody to an HA-epitope (HA.11) was obtained from Santa Cruz. A mouse monoclonal antibody to an HA-epitope (clone 12CA5) was purchased from Boehringer Mannheim. Rabbit polyclonal antibodies to ULK1 (A7481) and Atg13 (SAB4200100) were from Sigma. Rabbit polyclonal antibodies to LC3 (PM036) and p62 (PM045) were acquired from Medical & Biological Laboratories (MBL). Rabbit polyclonal antibodies to FIP200, p53, and COP1 were generated using bacterially produced polypeptides in our laboratory. A rabbit polyclonal antibody to Atg101 was provided by Dr. Noboru Mizushima.

### Split GFP assay

GFP (YFP) was split into two domains, N-terminal (YN: amino acids 1–154) and C-terminal (YC: amino acids 155–238). Each domain was fused to two molecules (full-length COP1 and FIP200 in this case), and transfected into cells as described above. GFP signals were observed using phase-contrast or fluorescence microscopy and measured with a flow cytometer. A human cDNA clone containing entire coding sequence of FIP200 was obtained from Kazasa DNA Research Institute (clone niumber: KIAA0203).

### Tumorigenicity assay

Cells (ca 10^6^) were subcutaneously injected into NOD-SCID mice. After 3 weeks or 2.5 months, mice were sacrificed and the size of the tumor was measured.

## Conclusion

In this study, we found the interaction between FIP200 and COP1. Ectopic expression of COP1 reduced one of the different forms of FIP200, suggesting that COP1 modulates FIP200-associated activities, which may contribute to a variety of cellular functions that COP1 is involved in.

## Authors' contributions

SK carried out the molecular biological studies. NI carried out the molecular cellular studies. AY performed the cellular analysis. NY-K and J-yK conceived of the study and participated in its design and coordination and drafted the manuscript. All authors read and approved the final manuscript.
